# A meta-analysis of the prognostic impact of tissue golgi protein 73 (tGP73) in hepatocellular carcinoma

**DOI:** 10.1186/s12876-023-03050-5

**Published:** 2023-11-17

**Authors:** Wei-Ming Yu, Guo-Wei Li, Ming-Geng Lou, Zheng-Yu Wu

**Affiliations:** Department of Hepatobiliary and pancreatic surgery, The First People’s Hospital of Fuyang District, Fuyang First Hospital Affiliated to Binjiang College of Zhejiang Chinese Medicine University, Hangzhou, China

**Keywords:** Tissue Golgi Protein 73, Hepatocellular Carcinoma, Prognosis, Meta-analysis

## Abstract

**Introduction:**

To date, an increasing number of studies have revealed that GP73 may have prognostic value in liver cancer. However, most of the studies evaluated serum GP73, and the results regarding the prognostic value of tGP73 in liver cancer are still controversial. Therefore, in this meta-analysis, we aimed to determine whether tGP73 has any prognostic value in patients with HCC.

**Materials and methods:**

Relevant publications were searched for in PubMed, EMBASE, OVID, the Cochrane Library, and the Web of Science databases up to March 2023. The hazard ratio (HR) or odds ratio (OR) with corresponding 95% confidence intervals (95% CIs) of eligible studies were assessed by fixed-effects or random-effects models. In addition, subgroup analyses were conducted to investigate the possible causes of heterogeneity, and publication bias analysis was also performed to assess the reliability of the meta-analysis results.

**Results:**

A total of 10 studies were included. These studies included 1569 HCC patients, and a meta-analysis was performed. The results of our meta-analysis showed that higher GP73 expression levels were significantly associated with poorer OS (HR = 1.87, 95% CI: 1.41–2.48, P < 0.0001, I^2^ = 58%). However, there was no significant correlation between high GP73 expression and disease-free survival (DFS) (HR: 1.43, 95% CI: 0.93–2.33, P = 0.100). In addition, abnormal GP73 expression was also related to higher tumour tissue differentiation grade (OR = 3.03, 95% CI = 2.01–4.57, P < 0.0001, I^2^ = 89%), later tumour stage (OR = 5.89, 95% CI = 2.31–14.99, P < 0.0001, I^2^ = 0%), vascular invasion (OR = 1.72, 95% CI = 1.12–2.64, P = 0.010, I^2^ = 0%), multiple tumours (OR = 2.44, 95% CI = 1.37–3.68, P = 0.001, I^2^ = 44%) and early postoperative tumour recurrence (OR = 1.92, 95% CI = 1.10–3.28, P = 0.020, I^2^ = 62%).

**Conclusions:**

The meta-analysis showed that the overexpression of GP73 may be related to a poor prognosis of HCC, and it may also have a predictive effect on the invasion and metastasis of HCC.

## Introduction

Hepatocellular carcinoma (HCC) is the fifth most common malignant tumour worldwide and the third leading cause of cancer-related death [1, 2]. Hepatectomy, liver transplantation and local ablation are radical treatments for patients with early liver cancer, while transcatheter arterial chemoembolization (tace), targeted therapy and immunotherapy are the most commonly used treatments for patients with advanced liver cancer who are not suitable for radical treatment [3–6]. Although remarkable progress has been made in early detection, diagnosis and treatment, the curative rate of HCC is still not satisfactory due to the high rate of recurrence or metastasis within 5 years after treatment [7].

Some prognostic indicators that have a negative impact on prognosis after treatment have been identified and include solid tumour size, tumour number, tumour differentiation, liver cirrhosis, vascular lymphatic infiltration, serum alpha-fetoprotein (AFP) levels and positron emission tomography (PET)/computed tomography (CT) results. Further prognostic indicators are sought to stratify patients with HCC more accurately and to determine the best treatment procedures, such as surgical resection or TACE treatment, as well as adjuvant therapy.

An increasing number of studies have shown that tissue GP73 (tGP73) is a new biomarker for hepatocellular carcinoma [8–12]. First, Chen et al. [13] reported that the high expression of tGP73 in hepatocellular carcinoma was significantly related to poor overall survival and disease-free survival. However, according to the study of Shan et al., the overall survival rate and disease-free survival rate of the high tGP73 group were significantly better than those of the low tGP73 group [14]. Additionally, according to Zhang et al., there is no statistical correlation between high tGP73 expression and disease-free survival (DFS) [15]. According to the above research results, the prognostic significance of tGP73 in surgically resected hepatocellular carcinoma is still controversial. Therefore, it is necessary to conduct a meta-analysis to comprehensively and systematically understand the value of tGP73 in the prognosis of patients after liver cancer surgery. In this study, we aimed to evaluate the prognostic significance of tGP73 in hepatocellular carcinoma (HCC).

## Materials and methods

### Search strategy

We performed a comprehensive literature search of articles through the following databases without date limitations: PubMed, Embase, OVID, The Cochrane Library and Web of Science databases. The search was conducted up to March 31, 2023. The main search terms included: (“Golgi protein 73”[Title/Abstract] OR “GP73” [Title/Abstract]) AND (“Hepatocellular Carcinoma*”[Title/Abstract] OR “Neoplasm*, Hepatic”[Title/Abstract]OR “Liver neoplasm*”[Title/Abstract] OR “Liver cancer*”[Title/Abstract] OR “Neoplasm*, Liver”[Title/Abstract] OR “Hepatic Neoplasm*”[Title/Abstract] OR “Cancer of Liver”[Title/Abstract] OR “Cancer*, Hepatocellular”[Title/Abstract] OR “Cancer of the Liver”[Title/Abstract] OR “Hepatic Cancer*”[Title/Abstract] OR “Cancer*, Hepatic”[Title/Abstract] OR “Carcinoma*, Hepatocellular”[Title/Abstract] OR “Liver Cell Carcinoma*”[Title/Abstract] OR “Carcinoma*, Liver Cell”[Title/Abstract] OR “Cell Carcinoma*, Liver”[Title/Abstract] OR “Hepatoma*”[Title/Abstract]). The reference list was also checked for relevant articles. This study is reported according to the Preferred Reporting Items for Systematic Reviewers and Meta-Analyses (PRISMA) guidelines [16].

### Inclusion and exclusion criteria

The inclusion criteria for selecting the studies for this meta-analysis were as follows: (1) HCC was confirmed by pathological examination; (2) the expression of GP73 in liver tissue samples of each patient was detected by immunohistochemistry analysis; (3) patients underwent primary curative surgical resection; and (4) the correlation of tissue GP73 with overall survival (OS) and/or disease-free survival (DFS) was reported. The exclusion criteria were as follows: (1) abstracts, letters, case reports, reviews or nonclinical studies; (2) patients who underwent neoadjuvant radiotherapy and chemotherapy before surgery; (3) sample size less than 10; and (4) studies with insufficient data for estimating hazard ratios (HRs) and 95% confidence intervals (CIs).

### Data extraction and quality assessment

The following data were extracted by two independent investigators (Weiming Yu and Minggeng Lou): first author, publication year, study region, sample size, age, sex, treatment method, cut-off value, follow-up period, clinicopathological features for HCC (HBV infection, serum AFP level, tumour differentiation, tumour stage, tumour size, tumour number, vascular invasion and recurrence status), and HR or OR with 95% CIs. Articles that could not be categorized based on title and abstract alone were retrieved for full-text review. If disagreement occurred, two investigators discussed and reached a consensus with a third investigator (Guowei Li). The Newcastle‒Ottawa Scale (NOS) was used to assess the quality of each of the included studies by two independent authors (Zhengyu Wu and Weiming Yu). The NOS consists of three parts: selection (0–4 points), comparability (0–2 points), and outcome assessment (0–3 points). NOS scores ≥ 6 were regarded as high-quality studies.

### Statistical analysis

All statistical analyses were conducted using Review Manager 5.3 (available from http://www.cochrane.org/). We directly obtained HR and 95% CI values from each study or estimated these data through survival curves. HR > 1 indicated that overexpression of GP73 in liver tissue is associated with worse overall survival (OS) and disease-free survival (DFS). Cochran’s Q test and Higgins I-squared statistic were used to assess the heterogeneity of the included trials. Both fixed-effects (Mantel‒Haenszel method) and random-effects (DerSimonian‒Laird method) models were used to calculate the pooled HRs or ORs and their 95% CIs. A P heterogeneity < 0.10 or I^2^ > 50% suggested significant heterogeneity in the literature, and a random-effects model was used. Otherwise, the fixed-effects model was adopted. Subgroup analysis was conducted to explore and explain the diversity (heterogeneity) among the results of different studies. Publication bias was assessed by Begg’s funnel plot. A p value of less than 0.05 was considered statistically significant.

## Results

### Study characteristics

**Fig. 1:**
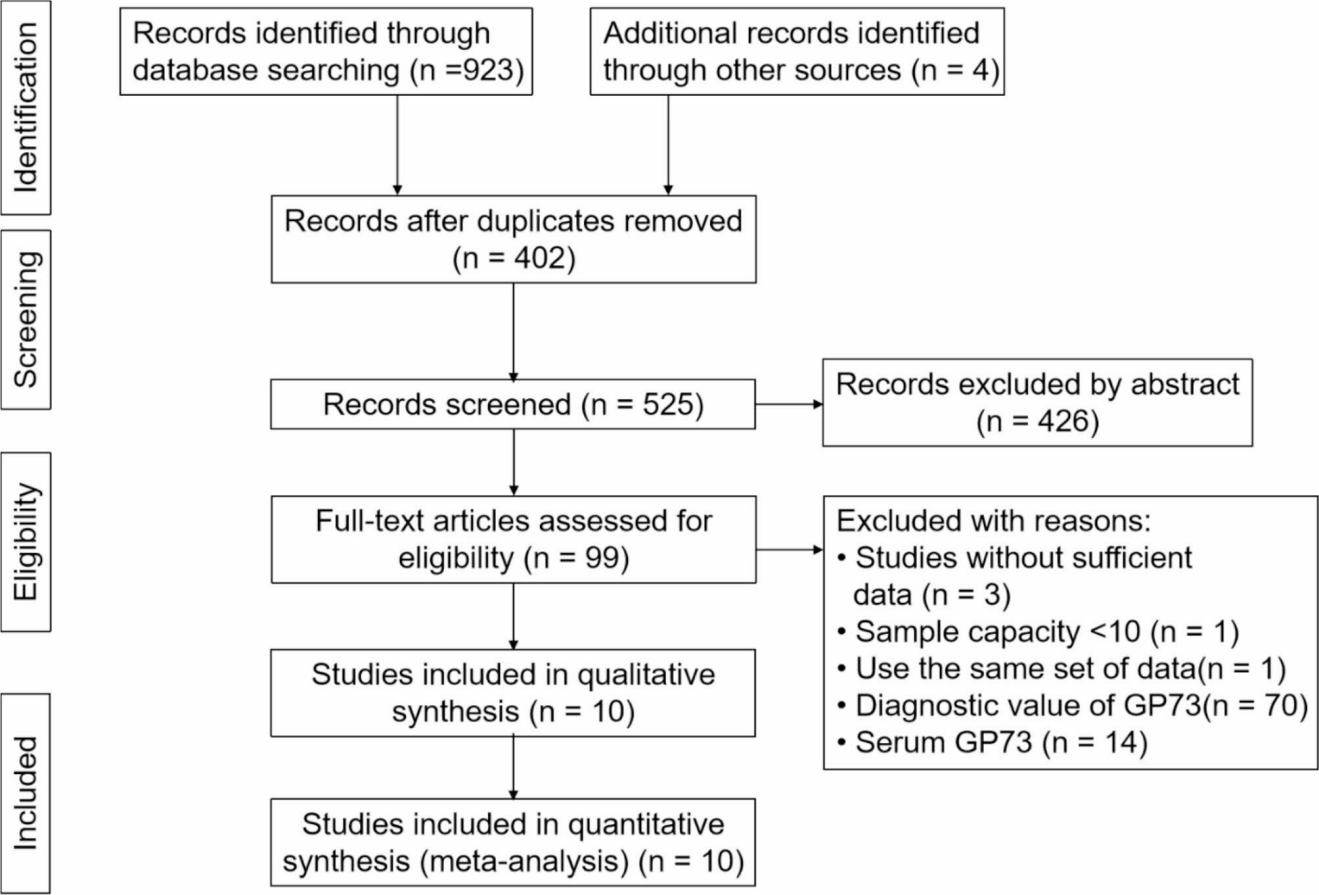
The flow diagram

A total of 927 articles were collected in the initial search. After careful inspection of these articles, 10 studies including 1569 patients published between 2013 and 2018 were finally enrolled in our meta-analysis. The detailed processes of study selection are shown in the flow diagram (Fig. 1). All of the studies that were obtained were from China. HRs and 95% CIs were extracted directly in 2 studies, and HRs were assessed by multivariate analysis. Both HRs and 95% CIs were estimated independently by survival curves from the remaining eight studies. The expression of GP73 in liver cancer tissues of all patients was determined using immunohistochemistry. All patients received radical surgery. In our meta-analysis, 9 studies demonstrated an association between tGP73 and OS, and 5 studies demonstrated an association between tGP73 and DFS. The characteristics of the enrolled studies are shown in Table 1.

**Table 1:** The basic characteristics of the enrolled studies

Author	Year	Study region	Sample size	Treatment	differentiation(Edmondson grade)	stage of tumour(TNM stage)	Cut-off value	OS HR(95% CI)	DFS HR(95% CI)	Method of extraction	follow-up periods(months)	NOS score
Bao et al. [8]	2013	China	75	Hepatectomy	I-IV	I-IV	20%	3.53(1.39–8.57)	NA	survival curve	20	6
Sai et al. [17]	2015	China	88	Hepatectomy	G1-G3	I-IV	25%	1.65(0.66–4.16)	NA	survival curve	60	7
Jiang et al. [10]	2016	China	90	Hepatectomy	I-II	NA	25%	1.7(0.92–3.16)	NA	survival curve	96	8
Yang et al. [11]	2017	China	64	Hepatectomy	G1-G3	NA	NA	NA	1.63(0.44–6.10)	survival curve	83	7
Mao et al. [12]	2018	China	54	Hepatectomy	G1-G3	NA	1.6 relative units	1.14(0.03–37.86)	1.2(0.30–4.87)	survival curve	67	8
Chen et al. [13]	2013	Taiwan	193	Hepatectomy	NA	I-III	150 scores	1.696(1.160–2.479)	1.765(1.100–2.026)	report	172	7
Shan et al. [14]	2013	China	62	Hepatectomy	NA	I-IV	NA	0.338(0.132–0.855)	0.485(0.242–0.972)	report	30	6
Dai(1) et al. [9]	2015	China	217	Hepatectomy	NA	NA	NA	2.52(1.56–4.09)	NA	survival curve	68	7
Dai(2) et al. [9]	2015	China	173	Hepatectomy	NA	NA	NA	3.76(2.26–6.27)	NA	survival curve	68	7
Ye(1) et al. [18]	2016	China	91	Hepatectomy	G1、G3	I-III	2 scores	1.59(0.84–3.02)	2.28(1.30-4.00)	survival curve	140	7
Ye(2) et al. [18]	2016	China	282	Hepatectomy	G1、G3	I-III	2 scores	1.79(1.28–2.50)	1.68(1.21–2.23)	survival curve	120	7
Li et al. [19]	2015	China	180	Hepatectomy	I-IV	NA	25%	1.96(1.18–3.27)	NA	survival curve	60	6

### tGP73 and OS in HCC

**Fig. 2:**
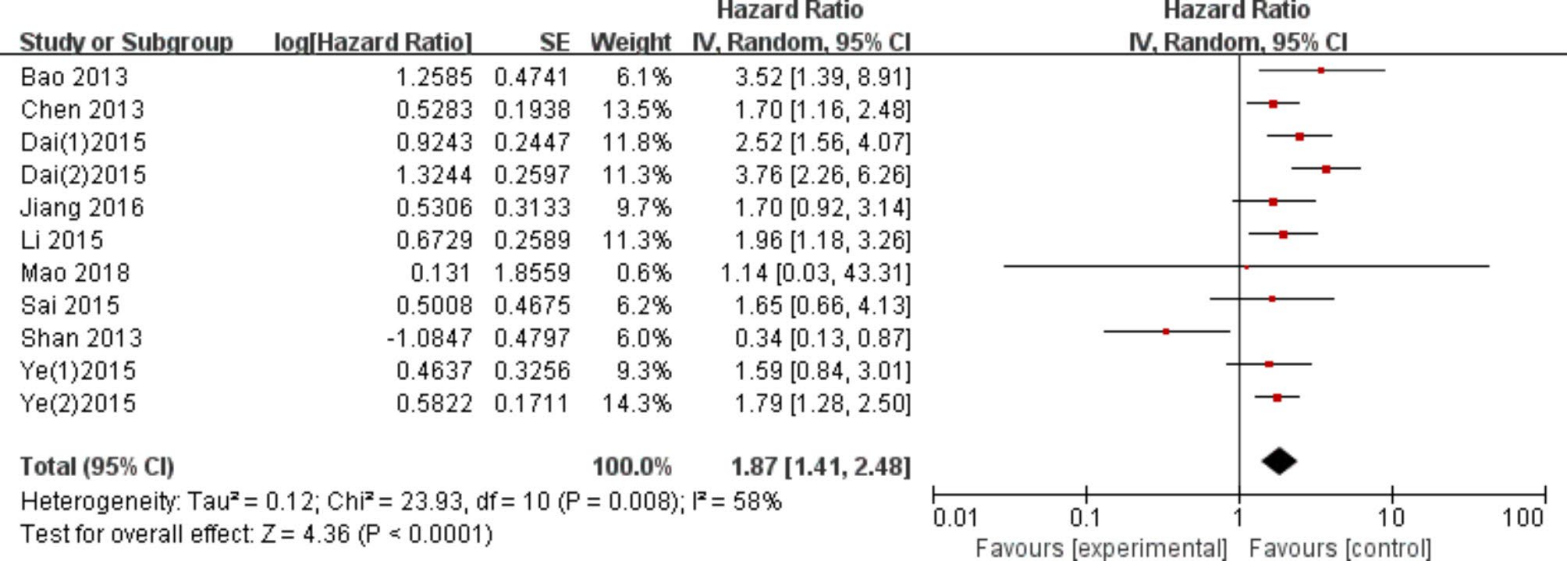
Association between tGP73 and overall survival of HCC

Nine studies evaluated the association between tGP73 and the overall survival of HCC. Due to the significant heterogeneity in the study (I^2^ = 58%, P = 0.008), we adopted a random effects model for analysis. The results clearly showed that HCC patients with tGP73 overexpression had a shorter OS, with a combined HR of 1.87 (95% CI: 1.41–2.48, P < 0.0001; Fig. 2).

### tGP73 and DFS in HCC

**Fig. 3:**
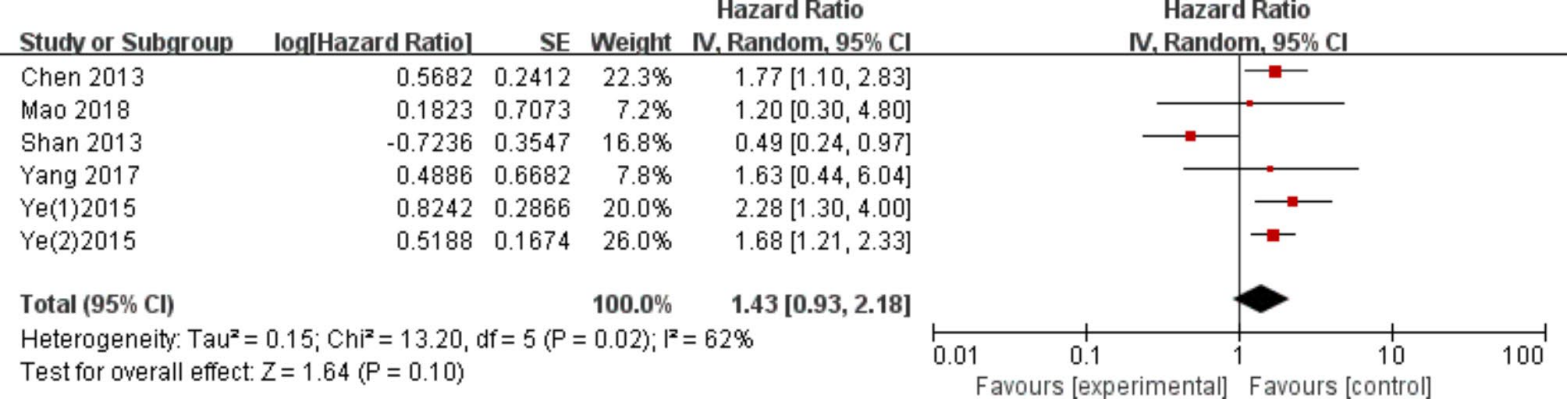
Association between tGP73 and disease-free survival of HCC

Five articles reported the association between tGP73 expression and DFS in HCC. Meta-analysis of these five studies showed no significant association between elevated tGP73 levels and DFS (HR: 1.43, 95% CI: 0.93–2.33, P = 0.100; Fig. 3). Since heterogeneity was evident in the study (I^2^ = 62%, p = 0.02), the random-effects model was applied to calculate the pooled HR.

### Relationship between tGP73 and clinicopathological characteristics of HCC

#### tGP73 and HBV

Five studies reported HBV infection (positive or negative), and the pooled results suggested that the overexpression of tGP73 had no correlation with HBV infection in HCC patients (OR = 1.31, 95% CI = 0.55–3.14, P = 0.540) (Table 2).

**Table 2:** Association between high tGP73 expression and clinicopathological features

Parameter	N of studies	N of patients	Effect model	OR (95% CI)	P value	Heterogeneity test	
						I^2^ (%)	P value
HBV (+/-)	5	451	Random	1.31(0.55,3.14)	0.540	58	0.050
AFP value (>400/≤400)	6	648	Random	0.88(0.60,1.28)	0.510	64	0.020
Edmondson grade (III-IV/I-II)	3	448	Random	3.03(2.01,4.57)	0.000	89	0.000
Vascular invasion (+/-)	4	491	Fixed	1.72(1.12,2.64)	0.010	0	0.400
TNM Stage (III–IV/I–II)	2	163	Fixed	5.89(2.31,14.99)	0.000	0	0.770
Liver cirrhosis	3	453	Fixed	1.03(0.68, 1.55)	0.900	6	0.350
Tumour number (multiple/single)	4	399	Fixed	2.44(1.37, 3.68)	0.001	44	0.150
Tumour size (≥ 3 cm/< 3 cm)	3	311	Fixed	0.92(0.55, 1.53)	0.750	0	0.690
Recurrence status	3	311	Random	1.92(1.10, 3.38)	0.020	62	0.070

CI – confidence interval, tGP73 – tissue Golgi protein 73, HBV – hepatitis B virus, N – number, OR – odds ratio

#### tGP73 and serum AFP levels

Serum AFP (> 400 or ≤ 400) was reported in 6 studies, and the pooled result showed no significant association between elevated tGP73 levels and serum AFP levels (OR = 0.88, 95% CI = 0.60–1.28, P = 0.510) (Table 2).

#### tGP73 and histological grade

Three studies reported histological grade (Edmondson grade III-IV or I-II), and the pooled results indicated that the overexpression of tGP73 was related to poor histological differentiation in HCC patients (OR = 3.03, 95% CI = 2.01–4.57, P < 0.0001) (Table 2).

#### tGP73 and vascular invasion

Vascular invasion (positive or negative) was reported in 4 studies, and the pooled results indicated that the group with overexpression of tGP73 had a higher incidence of vascular invasion (OR = 1.72, 95% CI = 1.12–2.64, P = 0.010) (Table 2).

#### tGP73 and tumour stage

Two studies presented data about tGP73 and tumour stage (TNM III-IV or I-II) in HCC patients. Our results indicated that the group with overexpression of tGP73 had a higher incidence of stage III and IV disease (OR = 5.89, 95% CI = 2.31–14.99, P < 0.0001) (Table 2).

#### tGP73 and liver cirrhosis

Three studies reported the association between tGP73 and the incidence of cirrhosis, and the pooled results indicated that tGP73 had no significant association with the incidence of liver cirrhosis (OR = 1.03, 95% CI = 0.68–1.55, P = 0.900) (Table 2).

#### tGP73 and tumour number

Four studies reported tumour number (multiple or single), and the combined results suggested that the incidence of multiple tumours was higher in HCC patients with overexpression of tGP73 (OR = 2.44, 95% CI = 1.37–3.68, P = 0.001) (Table 2).

#### tGP73 and tumour size

Tumour size (≥ 3 cm/< 3 cm) was reported in 3 studies, and the pooled results indicated that the tGP73 level had no correlation with tumour size (OR = 0.92, 95% CI = 0.55–1.53, P = 0.750) (Table 2).

#### tGP73 and tumour recurrence

Tumour recurrence was reported in three studies, and the pooled results suggested that the tGP73 overexpression group had a higher recurrence incidence (OR = 1.92, 95% CI = 1.10–3.38, P = 0.020) (Table 2).

### Subgroup analysis

We also conducted a subgroup analysis based on the HR extraction method, sample size and follow-up period to investigate the correlation between GP73 expression and OS (Table 3). In the survival curve subgroup analysis results of the study based on the HR extraction method, the combined HR was 1.93 (95% CI: 1.63–2.27, p < 0.0001). However, in the pooled data of the directly reported study, there was no significant correlation between high GP73 expression and OS (HR = 1.35, 95% CI: 0.95–1.92, p = 0.090). Additionally, in the subgroup analysis results of the study based on the sample size, the HR obtained from more than 100 studies was 2.09 (95% CI: 1.73–2.53, p < 0.0001). However, in studies with fewer than 100 cases, the combined HR was 2.16 (95% CI: 1.82–2.56, p = 0.020). Similarly, when these studies were analysed based on the follow-up period, the results showed that the prognosis of studies with a longer follow-up period (≥ 80 months) was significantly worse (HR = 1.72, 95% CI: 1.38–2.14, p < 0.0001). The same results were also observed in studies with a short follow-up period (< 80 months) (HR = 2.23, 95% CI: 1.74–2.88, p < 0.0001).

**Table 3:** Results of subgroup analysis for the effect of tGP73 on HCC overall survival

Parameter	N of studies	Effect model	Amalgamative HR(95% CI)	P value	Heterogeneity test	
					I^2^ (%)	P value
Overall	11	Random	1.87(1.41–2.48)	0.000	58	0.008
HR extraction method						
report	2	Random	1.35 (0.95, 1.92)	0.090	90	0.002
survival curve	9	Fixed	1.93 (1.63, 2.27)	0.000	14	0.320
Sample size						
≥ 100	5	Fixed	2.09 (1.73, 2.53)	0.000	48	0.100
<100	6	Random	2.16 (1.82, 2.56)	0.020	62	0.020
Duration of follow-up(months)						
≥ 80	4	Fixed	1.72(1.38, 2.14)	0.000	0	0.990
<80	7	Random	2.23 (1.74, 2.88)	0.000	72	0.001

CI – confidence interval, tGP73 – tissue Golgi protein 73, HCC – hepatocellular carcinoma, HR – hazard ratio, N – number

### Publication Bias

**Fig. 4:**
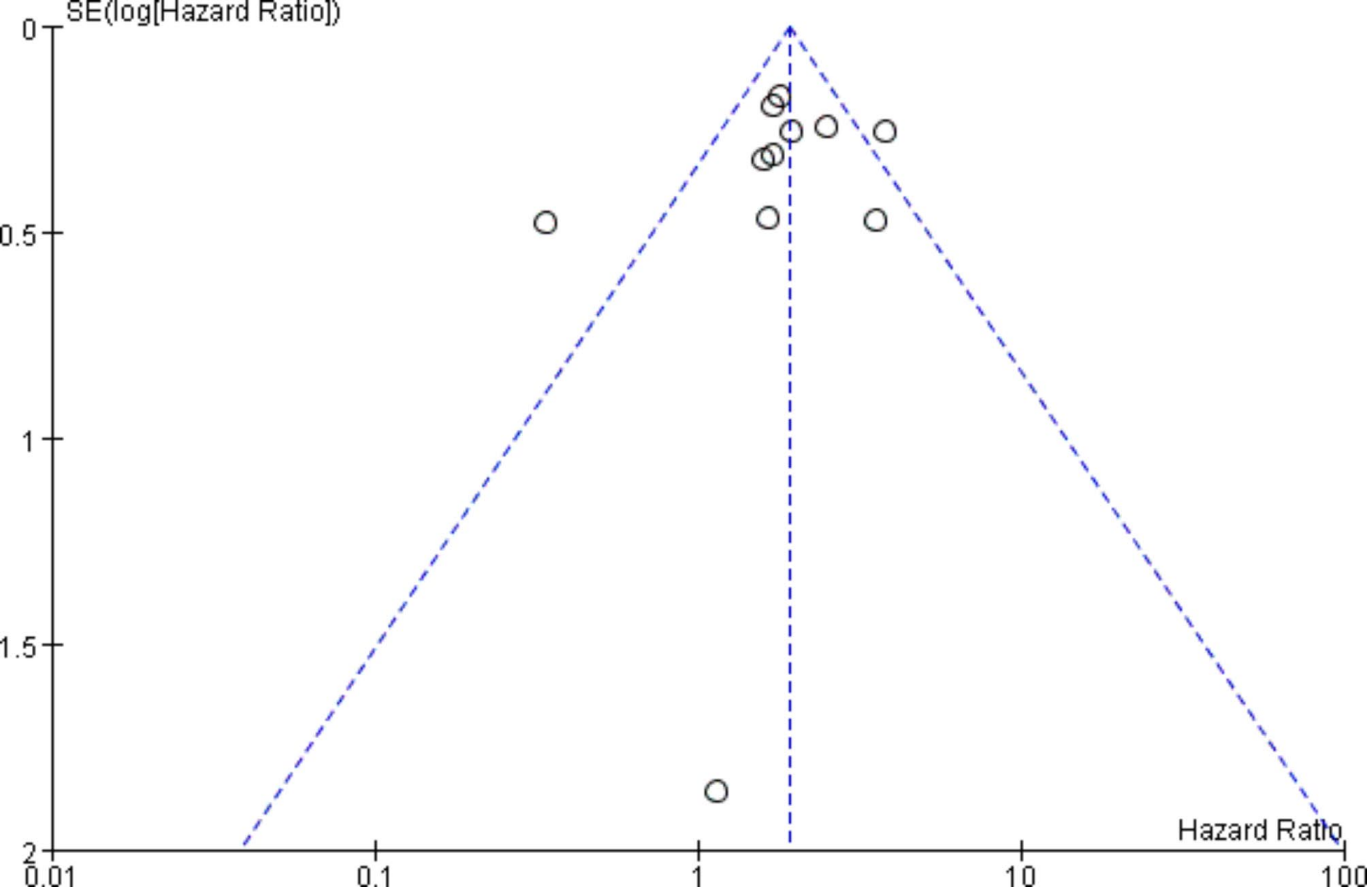
Funnel plot for assessing publication bias in OS

The function of publication bias analysis is to assess the reliability of the meta-analysis results, especially those with statistical significance [20]. Based on the OS data, publication bias was evaluated by Begg’s funnel plot. The shape of the funnel plot was visually roughly symmetrical (Fig. 4), and no significant evidence of publication bias was obtained.

## Discussion

GP73, a new type of 73 kDa human Golgi protein, was discovered by Kladney et al. [21] in 2000 through subtractive hybridization of liver cDNA libraries of patients with acute adult giant cell hepatitis and normal subjects. In normal human liver tissue, GP73 is mainly expressed in bile duct epithelial cells, with little or no expression in liver cells. The significant increase in GP73 expression may be the result of hepatocellular viral infections (HBV and HCV) or nonviral causes (alcoholic liver disease, autoimmune hepatitis, cirrhosis, and liver malignancies) [21–23], suggesting that GP73 expression may respond to different pathogenic mechanisms, such as acute and chronic hepatitis, cirrhosis, and HCC. Shan et al. [14] found that GP73 expression levels were higher in hepatocellular adenomas and focal nodular hyperplasia than in corresponding peritumoral liver tissues, and GP73 expression levels were also higher in the HCC group than in corresponding nontumor tissues, benign liver tumours and normal liver tissues. These findings indicate that during the progression of benign liver disease to precancerous lesions and HCC, the expression levels of GP73 increased. GP73 increased gradually with as the severity of liver injury increased. Serum GP73 levels were significantly higher in HCC patients than in healthy, chronic hepatitis, and cirrhosis control individuals [17, 22]. This difference may be due to the different degrees of differentiation of HCC cells with different protein synthesis and secretion capabilities or may be caused by the different regulatory mechanisms of GP73 in benign and malignant liver diseases.

Previous studies have shown that serum GP73 has important value in the early diagnosis and prognosis of liver cancer [24–29]. However, there are few studies on the relationship between the expression of GP73 in tissue and the prognosis of hepatocellular carcinoma (HCC), and no consensus has been reached thus far. Therefore, the purpose of this meta-analysis was to evaluate the prognostic effect of tissue Golgi protein 73 (tGP73) on hepatocellular carcinoma and to clarify the relationship between tGP73 and the clinicopathological features of the tumour.

In fact, the prognostic significance of tGP73 for overall survival and disease-free survival in patients undergoing hepatectomy is still controversial. Yang et al. [11] reported that the overexpression of tGP73 is a key risk factor for overall survival and disease-free survival in patients undergoing hepatectomy. However, Shan et al. [14] reported that the overall survival and disease-free survival of the high tGP73 group were significantly better than those of the low tGP73 group. In our meta-analysis, we included a number of studies that first established tGP73 as an important prognostic factor for HCC through univariate Cox regression analysis of all variables. Kaplan‒Meier survival analysis of tGP73 further confirmed that the survival curves corresponding to different tGP73 levels were different, suggesting the reliability of tGP73 as a prognostic factor. Moreover, multivariate Cox regression analysis further demonstrated that tGP73 expression was an independent prognostic marker for the survival of HCC patients undergoing hepatectomy. Our meta-analysis included the prognosis of 1569 patients with hepatocellular carcinoma from 10 individual studies. Nine studies have shown a correlation between the expression of tGP73 and OS in hepatocellular carcinoma. The combined HR of 1.87 (95% CI: 1.41–2.48, P < 0.0001; Fig. 2) showed that the overall survival time of HCC patients with overexpression of tGP73 was shorter after surgery. Five studies provided data related to the expression of tGP73 and DFS, and the combined HR was 1.43 (95% CI: 0.93–2.33, P = 0.100; Fig. 3), indicating that there was no significant correlation between tGP73 and disease-free survival in HCC patients undergoing hepatectomy.

The role of GP73 in the development of HCC and its involvement in tumorigenesis remain unclear. There is abundant evidence that GP73 overexpression is closely related to the aggressiveness and metastasis of tumours. Sun et al. [30] reported that GP73 levels are closely related to tumour size, venous infiltration and tumour differentiation, suggesting that GP73 can enhance tumour invasion and metastasis. Bao et al. [8] found that GP73 expression was positively correlated with epithelial-mesenchymal transformation (EMT), and high levels of GP73 were correlated with Edmondson grading, vascular invasion and TNM staging. These findings indicate that GP73 is involved in regulating EMT to promote the invasion of HCC. Chen et al. [31] showed that upregulation of GP73 can promote the proliferation and migration of mouse HCC cell lines and the development of tumours. Ye et al. [18] reported that GP73 drives HCC metastasis by regulating EGFR/RTK cell surface recycling. According to the report of Jiang et al. [10], GP73 may play an important role in liver cancer metastasis by inducing EMT. Additionally, the expression level of GP73 in HCC tissues is higher than that in paired noncancer tissues, and the expression level of GP73 in HCC tissues with metastasis is higher than that in the primary tumour. The survival rate of patients with high GP73 expression is significantly lower than that of patients with low GP73 expression, which is consistent with the research results of Chen et al. [13]. This suggests that the high expression levels of GP73 in tumour tissues are associated with metastasis and a low survival rate. The research data of Dai et al. [9] showed that GP73, as an oncoprotein, is involved in the occurrence and development of liver cancer, and it can play a key role in controlling liver cancer invasion and angiogenesis by inducing activation of the NF-κB signalling pathway. In summary, GP73 may be a potential biomarker for HCC prognosis.

From our results, the overexpression of tGP73 can significantly predict the overall survival of patients undergoing hepatectomy. Additionally, patients with high tGP73 expression tended to have shorter disease-free survival, but the difference was not statistically significant, which may be due to the small number of included studies. However, studies by Mao and Ye have also confirmed that high expression of tGP73 has a negative effect on overall survival and disease-free survival in patients undergoing hepatectomy [12, 18]. Taken together, these data collectively suggest that high expression of tGP73 is an independent risk factor for OS and DFS in HCC patients undergoing hepatectomy.

Additionally, we also analysed the correlation between the expression of tGP73 and tumour clinicopathological characteristics (including HBV infection, serum AFP level, degree of tissue differentiation, vascular invasion, tumour TNM stage, liver cirrhosis, tumour number, tumour size, and tumour recurrence), which are highly correlated with the aggressiveness and metastasis of HCC [32, 33]. The results showed that the overexpression of tGP73 was significantly correlated with higher tumour tissue differentiation grade, later tumour stage, vascular invasion, multiple tumours, and early tumour recurrence after surgery, which are consistent with the findings of Mao et al. [12] and Bao et al. [8].

When significant heterogeneity was found in the study of the correlation between GP73 expression and OS, we further carried out subgroup analysis. First, we carried out stratified analysis according to the HR extraction method (including estimation or direct extraction by survival curve) and subgroup analysis according to sample size (≥ 100 or < 100) and follow-up time (≥ 80 months or < 80 months). The results suggest that the HR extraction method, sample size and follow-up time may be the source of high heterogeneity.

Our meta-analysis has some limitations. First, the included studies are retrospective, which leads to some biases that were inevitable. Second, all the studies in our meta-analysis were from China, and all patients were treated with surgery. Thus, whether our conclusions are applicable to people of other races or receiving other treatments remains to be studied further. Third, although the HR extraction method, sample size and follow-up time have been indicated to be the three sources of heterogeneity, other factors such as baseline characteristics of the study population and changes in the critical value of tGP73 may also lead to a high degree of heterogeneity in the current study.

Despite the above limitations, our meta-analysis shows that the overexpression of tGP73 is closely related to the poor prognosis of HCC patients undergoing surgery. In addition, our results indicate that the overexpression of tGP73 is closely related to the clinicopathological features that represent the invasiveness and metastasis of HCC, including higher grade of tumour differentiation, later tumour stage, vascular invasion, multiple tumours and early postoperative recurrence. There is a difference between serum GP73 and tissue GP73. Since most HCC patients suffer from LC, sGP73 is secreted from tumour tissues and cirrhotic tissues, and the overall level of sGP73 is not enough to accurately express the true level of GP73 in tumour tissues. Therefore, tissue results are superior to serum results in demonstrating the prognosis of HCC [14]. However, liver biopsy is more invasive for patients, and the detection of serum GP73 is more convenient, which may hinder the use of tGP73 in clinical practice. Additionally, liver fibrosis and viral infection can also upregulate tGP73 in liver tissue. Therefore, the efficacy of tGP73 in predicting the prognosis of HCC remains to be discussed. Nevertheless, our study demonstrates that tGP73 expression remains a promising prognostic marker that can contribute to the clinical decision-making process for the treatment and prognosis of hepatocellular carcinoma. In the future, a larger sample of prospective studies is needed to further evaluate the relationship between tGP73 and the prognosis of hepatocellular carcinoma.

## Data Availability

The datasets used during the current study are available from the corresponding author on reasonable request.
